# Quantification of Chlorogenic Acid and Vanillin from Coffee Peel Extract and its Effect on α-Amylase Activity, Immunoregulation, Mitochondrial Oxidative Stress, and Tumor Suppressor Gene Expression Levels in H_2_O_2_-Induced Human Mesenchymal Stem Cells

**DOI:** 10.3389/fphar.2021.760242

**Published:** 2021-11-02

**Authors:** Heba Khalil Alyahya, Pandurangan Subash-Babu, Ahmad Mohammad Salamatullah, Khizar Hayat, Nawal Albader, Mohammed Saeed Alkaltham, Mohammed Asif Ahmed, Shaista Arzoo, Mohammed Bourhia

**Affiliations:** ^1^ Department of Food Science and Nutrition, College of Food and Agricultural Sciences, King Saud University, Riyadh, Saudi Arabia; ^2^ Laboratory of Chemistry‐Biochemistry, Environment, Nutrition and Health, Faculty of Medicine and Pharmacy, Hassan II University, Casablanca, Morocco

**Keywords:** coffee peel, polyphenol, flavonoid, chlorogenic acid, antioxidant, DNA integrity

## Abstract

**Background:** Polyphenols and flavonoid-rich foods help in arresting reactive oxygen species development and protecting DNA from oxidative damage. Coffee peel (CP) preparations are consumed as beverages, and their total polyphenol or flavonoid content and their effect on oxidative stress–induced human mesenchymal stem cells (hMSCs) are poorly understood.

**Method:** We prepared hot water extracts of CP (CPE) and quantified the amount of total polyphenol and flavonoid using HPLC analysis. In addition, CPE have been studied for their α-amylase inhibitory effect and beneficial effects in oxidative stress–induced hMSCs.

**Results:** The obtained results show that the availability of chlorogenic acid, vanillin, and salicylic acid levels in CPE is more favorable for enhancing cell growth, nuclear integrity, and mitochondrial efficiency which is confirmed by propidium iodide staining and JC-1 staining. CPE treatment to hMSCs for 48 h reduced oxidative stress by decreasing mRNA expression levels of LPO and NOX-4 and in increasing antioxidant CYP1A, GSH, GSK-3β, and GPX mRNA expressions. Decreased pro-inflammatory (TNF-α, NF-κβ, IL-1β, TLR-4) and increased tumor suppressor genes (except Bcl-2) such as Cdkn2A, p53 expressions have been observed.

**Conclusions:** The availability of CGA in CPs effectively reduced mitochondrial oxidative stress, reduced pro-inflammatory cytokines, and increased tumor suppressor genes.

## Introduction

Globally, coffee is the most frequently consumed drink, and the increasing interest in coffee consumption has led the manufacturers to produce more varieties of coffee and is still progressive (Song et al., 2019). In the year 2019, the global green coffee production reached 10.03 billion kilograms (FAOSTAT, 2019). The large-scale production of coffee powder leads to an enormous amount of coffee peel (CP) waste (Murthy and Naidu, 2012; Prihadi et al., 2020). CP contains high fiber, and it has been identified with antioxidant, antiallergic, antihypertensive, and antimicrobial potential (Prihadi et al., 2020). The phytochemicals present in the CP are caffeine, tannin, polyphenol, pectin, and monosaccharide and disaccharide compounds (Janissen and Huynh, 2018). In coffee, the major beneficial bioactive compounds are caffeine and various polyphenols such as chlorogenic acid (CGA), ferulic acid, sinapic acid, gallic acid, and quinic acid (Hečimović et al., 2011; Durak et al., 2014). The main component of the phenolic fraction of the green coffee bean is CGA (Farah and Donangelo, 2006). CGA possesses a variety of health benefits such as reducing the risk of diabetes, cancer, and liver disease and protecting against Parkinson’s disease (Bhupathiraju et al., 2013; Cano-Marquina et al., 2013).

Antioxidant compounds are majorly obtained from coffee and they contribute to the total dietary antioxidant capacity (Fujioka and Shibamoto, 2006). CGA is the main antioxidant compound in coffee which provides an activity similar to ascorbic acid (Pari et al., 2010). In addition, CGA prevents oxidized LDL and H_2_O_2_-induced oxidative stress and effectively scavenges the free radicals (superoxide, hydroxyl, and peroxynitrite) and protects DNA from oxidative damage (Cha et al., 2014). Meantime, excessive intake of coffee leads to higher bioavailability of CGA, which inhibits endothelial cells and cancer cell growth and angiogenesis; inversely, inhibition of angiogenesis and neovascularization cause impaired cell growth, tissue ageing, and functional disability (Wang et al., 2021). The present study emphasizes on CP, which contains high fiber and limited CGA, to be used as an antioxidant, antiallergic, antihypertensive, and antimicrobial agent (Prihadi et al., 2020).

CPs containing biomolecules need to be considered as a major antioxidant agent to protect against reactive oxygen species (ROS)–induced oxidative stress in human mesenchymal stem cells (hMSCs) during tissue repair or tissue regeneration therapy. Human MSCs play a major role in regenerative and immunomodulatory properties due to their multipotent differentiation potential (Denu and Hematti, 2016). Human MSCs are more sensitive to oxidative stress, and excessive ROS or exogenous H_2_O_2_ can impair self-proliferation and multilineage capacity (Choo et al., 2014). They are implanted to injured tissues and contribute to tissue repair with suppressing inflammatory rejection (Di Nicola et al., 2002). The loss of transplanted MSCs at the ischemic site is a major problem due to the loss of chemokine receptors, after the generation of ROS at the graft site (Honczarenko et al., 2006). ROS are initially generated from the mitochondrial complex (I & III) and NOX4 during hMSCs differentiation (Devine et al., 2001). Excessive ROS react and damage the biomolecules, especially altering the integrity of genomic DNA, which is critical for cellular proliferation and functions (Kobayashi et al., 2012).

The present study aimed to quantify the concentration of CGA, vanillin, and salicylic acid (SA) in CP extract (CPE) using HPLC. Furthermore, the potential of CPE on α-amylase inhibitory effect, bioefficacy in hMSCs *via* analyzing the inhibitory effect of cell and nuclear damage, mitochondrial membrane polarization, oxidative damage, and immunomodulation-related gene expression levels in H_2_O_2_-induced oxidative stressed hMSCs have been explored.

## Materials and Methods

### Raw Material

*Coffea arabica* was obtained from the Jazan region in Fifa Mountains (1800 m above sea level), Kingdom of Saudi Arabia. Fresh whole coffee fruits were selected, washed, dried in the sun, and saved in a dry place until their extraction. CP was removed (hulled) from the whole coffee fruit and ground by using a coffee grinder (SF Stardust, PCP-R400065, Japan). Finally, the ground CP was sieved with stainless steel (0.5 mm) wire mesh to ensure a consistent powder size and kept at room temperature (25°C) for further extractions.

### Preparation of the Extracts

CP was weighed (0.5, 0.75, 1, and 1.25 g) and then mixed with 10 ml water (solvent). Then the samples were boiled and stirred using a heating magnetic stirrer at 100°C for 10 min. Furthermore, the samples were allowed to rest for 10 min at room temperature and then centrifuged at 3000 *g* for 10 min using a fixed rotor centrifuge (Thermo Fisher Scientific, MA, United States). Finally, the samples were filtered using a filter paper (Whatman 9.0 cm). The obtained CPE was labeled properly, such as 0.5 g as CPE-1, 0.75 g as CPE-2, 1 g as CPE-3, and 1.25 g as CPE-4, and were kept under refrigeration at 4°C until analysis.

### Total Polyphenol Content

The total polyphenol content (TPC) of the CPE was determined according to the method described by Hayat et al. (2011). 25 μl of the CPEs (CPE-1, CPE-2, CPE-3, and CPE-4) was mixed with 1500 μl distilled water and 125 μl Folin–Ciocalteu reagent (0.2%) and allowed to stand for 1 min. After that, 375 μl of Na_2_CO_3_ (20% w/v) and 475 μl distilled water were added. Finally, the mixture was allowed to rest at room temperature for 30 min. The absorbance was measured at 760 nm using a spectrophotometer (JascoV-630 Spectrophotometer, United States). A blank was prepared without the extract. The TPC was expressed as a gallic acid equivalent per gram dry weight of the sample (mg GAE/g DW).

### Total Flavonoid Content

The total flavonoid content (TFC) was determined as described by Hayat (2020). 250 μl of CPEs (CPE-1, CPE-2, CPE-3, and CPE-4) was mixed with 1000 μl of distilled water. Then, 75 μl of each NaNO_2_ (5%) and AlCl_3_ (10%) was added and incubated at room temperature for 5 min. Then, 500 μl of NaOH (1 M) and 600 μl of distilled water were added. The absorbance was recorded with a spectrophotometer (JASCO V-630 Spectrophotometer, United States) at 510 nm. A blank was prepared without the extract, and each sample was repeatedly analyzed six times (*n* = 6) to get mean ± SD. The TFC was expressed as a catechin equivalent per gram dry weight of the sample (mg CE/g DW).

### Quantification of Phenolic Compounds in Coffee Peel Extracts Using HPLC

The quantification of phenolic compounds (tannic acid, CGA, caffeic acid, resorcinol, vanillin, 1,2-dihydroxybenzene, salicylic acid, acetyl salicylic acid, 3,5-dinitrosalicylic acid, and quercetin) in CPE(s) was determined using HPLC according to the method of Santos et al. (2014). The CPEs (CPE-1, CPE-2, CPE-3, and CPE-4) were separated by using the Shimadzu HPLC system, prominence (Kyoto, Japan) equipped with a LC-20AB binary pump and a variable Shimadzu SPD-10A UV-Vis detector. The column used was Zorbax SB-C18 (250 × 4.6 mm, 5 µm) (Agilent, Santa Clara, CA, United States) and the mobile phase consisted of (0.1% formic acid, A) and MeOH (0.1% formic acid, B). The gradient program was the following: 0 min, 5% B; 4 min, 5% B; 20 min, 73% B; 50 min, 95% B; 57 min, 1% B; 58 min, 1% B; and 60 min, 5% B, with a low rate of 0.7 ml/min. The injection volume was 10 µl, and the detector was set at 280 nm. Compounds were identified by comparing their retention time with those of the standard. All samples were analyzed in duplicates.

### Carbohydrate Hydrolysis Enzymatic α-Amylase Assay

The carbohydrate hydrolysis enzymatic α-amylase (α-A) assay was performed according to the procedure suggested previously by Subramanian et al. (2008) and Hasenah et al. (2006). The controlled sample contained 150 μl of α-A enzyme solution and 150 μl of distilled water. The sample mixture was prepared by adding 150 μl of α-A to 150 μl of extract (CPE-1, CPE-2, CPE-3, and CPE-4). Likewise, the positive control was prepared with 150 μl of 1 mM acarbose (dissolved in 1% DMSO (dimethylsulfoxide)) and 150 μl of α-A. The blank mixture was prepared by dissolving 150 μl of distilled water with 150 μl of 20 mM phosphate buffer (pH 6.9) without α-A and the extract. Each tube was gently mixed and incubated at 37°C for 10 min. Thereafter, 150 μl of 0.5% starch solution was added to initiate the reaction, and the tubes were incubated at 37°C for 30 min. Then, 300 μl of dinitrosalicylic acid was added to stop the reaction, and the tubes were placed in a water bath at 100°C for 10 min. Lastly, all tubes were cooled to room temperature and then diluted with 2 ml of distilled water. The absorbance was determined at 540 nm (JASCO V-630 Spectrophotometer, United States). The percentage of amylase inhibitory activity of each sample was calculated by using the following equations:

$$\%\;\text{Inhibition}=Adjusted\;control-\frac{Adjusted\;sample}{Adjusted\;control}X\;100.$$ (1)

### In Vitro Cell Culture Method and Materials

Human mesenchymal stem cells (hMSCs) have been obtained from the American type culture collection (ATCC, Manassas, VA, United States). DMEM (Dulbecco’s Modified Eagle medium), EDTA (ethylenediaminetetraacetic acid), and trypsin were purchased from Gibco (Paisley, United Kingdom). Cell culture materials such as FBS (fetal bovine serum) and penicillin–streptomycin were obtained from Hyclone Laboratories, United States. MTT [3-(4,5-dimethylthiazol-2-yl)-2,5-diphenyltetrazolium bromide], propidium iodide, JC-1 stain, and all other chemicals related to a molecular biology experiment have been purchased from Sigma-Aldrich (St. Louis, MO, United States). The cell to cDNA synthesis kits and SYBR green PCR master mix were purchased from QIAGEN (Hilden, Germany).

#### Human Mesenchymal Stem Cell Culture

Human mesenchymal stem cells (hMSCs) were cultured using DMEM added with 10% FBS and 100 U/ml penicillin–streptomycin at 37°C in a humidified 5% CO_2_ incubator. According to the experimental design and need, hMSCs were seeded in 96-well (1 ×10^4^ cells/well) or 24-well (2 ×10^4^ cells/well) plates containing DMEM with 10% FBS at 37°C and 5% CO_2_ in humidified air. After visual confirmation of 80% confluence under an inverted microscope, the culture was used for the experiments.

#### Cytotoxicity Analysis

Human mesenchymal stem cells (hMSCs) were cultured in a 96-well culture plate (1 × 10^4^ cells/well) and allowed to adhere overnight to the growth medium. After discarding the medium, a culture medium containing an increasing concentration (1, 2, 4, 8, 16, 32, and 64 mg/ml) of CPEs, namely, CPE-1, CPE-2, CPE-3, and CPE-4, was added to each well, and the cells were incubated for 48 h; control cells were treated with vehicle alone. After completion of 48 h, the cells were added with 20 µl/well of MTT [3- (4, 5-dimethylthiazol-2- yl)-2, 5-diphenyltetrazolium bromide] at a concentration of 5 mg/ml in DMSO and incubated at 37°C for an additional 4 h. At the end of incubation, the supernatant solution was removed gently without disturbing the formed purple formazan crystals. The crystal was dissolved in 100 µl of 100% DMSO using a multiwell plate shaker. The absorbance of the solution was measured at 570 nm using a microplate reader (Thermo Scientific). Cell proliferation (%) was calculated by the following equation: (absorbance of the sample/mean absorbance of the control) × 100.

### Experimental Design

Bioefficacy and oxidative stress inhibition capacity of CPE on normal and H_2_O_2_-induced oxidative stressed hMSCs were examined. Two sets of hMSCs were cultured in 24-well plates and treated with 4 mg/ml of CPEs, namely, CPE-1, CPE-2, CPE-3, and CPE-4, for 48 h, respectively. After incubation, one group was kept for normal observation, and the other group of hMSCs were treated with 10 μM of H_2_O_2_ for 30 min. CGA was used as a reference control in both the experimental sets. After incubation, all the grouped cells were used for propidium iodide staining, JC-1 staining, and gene expression analysis.

#### Propidium Iodide Staining Assay for Nuclear Damage

Cellular morphology for characteristic nuclear damage, pyknosis, or apoptotic morphological changes after hMSCs were treated with CPE-1, CPE-2, CPE-3, and CPE-4 (with or without H_2_O_2_) was determined using propidium iodide (PI) staining analysis under inverted fluorescence microscopy as described by Leite et al. (1999).

#### Assay of Mitochondrial Membrane Potential (Δψ_m_) by JC-1 Dye Staining

The mitochondrial membrane potential (Δψ_m_) was determined using the JC-1 dye to assess mitochondrial efficiency in the vehicle control, and CPE-1–, CPE-2–, CPE-3–, and CPE-4–treated hMSCs (with and without H_2_O_2_). Briefly, the JC-1 staining solution (mixed with equal volumes of the culture medium) was added to experimental hMSCs and incubated for 20 min in the dark at 37°C. After incubation, the unbound JC-1 dye was gently removed by washing with 200 μl of JC-1 staining wash buffer at 4°C, repeatedly for two times. Then, the accumulation of J-aggregates against JC-1 staining was observed under a florescence microscope.

#### Quantitative Real-Time PCR Analysis

Vehicle control; CPE-1–, CPE-2–, CPE-3–, and CPE-4–treated hMSCs (with and without H_2_O_2_); total RNA; and cDNA were synthesized using a Fastlane^®^ Cell cDNA kit using qPCR. mRNA expression levels of oxidative stress (LPO, NOS, and CYP1A), antioxidants (GSH, GSK-3β, and GPx), pro-inflammatory cytokines (TNF-α, NF-κb, IL-1β, and COX-2), and tumor suppressor (cdkn2a, p53, and BCL-2)–related genes, and the reference gene, β-actin, have been analyzed according to the method of Yuan et al. (2006). The amplification values (ΔCt) have been calculated by the difference between Ct (treated) and Ct (control). The gene expressions were plotted using the expression of 2^−ΔΔCt^ value.

### Statistical Analysis

All the experiments were performed in triplicates, and the data were presented as mean values ± SD (standard deviation). SAS software (version 9.2, 2000–2008; SAS Institute Inc., Cary, NC, United States) was used to analyze the differences among the groups by one-way analysis of variance (ANOVA), and if significant differences were found, then the Duncan's multiple range test was conducted at a confidence interval of 95% (*p* < 0.05).

## Results and Discussion

Recently, there has been a surge of interest in phenolic compounds extracted from plant materials (Kähkönen et al., 1999; Tapiero et al., 2002; Murthy and Naidu, 2012). They are secondary metabolites that occur naturally in plants and are generally intricate in defense against oxidative stress or aggression by pathogens. Considerable populations in Europe and the Arab regions consume hot water–boiled dried CP powder as an energy drink or refreshing drink as a replacement for coffee (Liang and Kitts, 2016; Farah and de Paula, 2019). The polyphenol compound CGA is the major antioxidant present in the coffee seeds and peel (Ameca et al., 2018). Meanwhile, CGA is a more thermolabile compound, and depending upon the different hot preparation or extraction methods, a considerable amount of CGA is lost (Liang and Kitts, 2016). The processes to extract phenolic compounds vary from plant to plant. Thoo et al. (2010) demonstrated that the extraction efficacy is dependent on various factors such as the method of extraction, extraction time, solvent concentration, solvent type, temperature, and solid-to-solvent ratio. During extraction of phenolic compounds, the key effect of the solid-to-solvent ratio was applied to adjust the solubility and equilibrium constant, resulting in a maximum yield of bioactive compounds at the optimum solid-to-solvent ratio (Tan et al., 2011) and avoidance of saturation effect, as well as a reduction of the solvent waste disposal cost (Ho et al., 2008). The current research, on the other hand, demonstrated the effectiveness of aqueous extraction which is also known as a nonpolluting solvent for obtaining phenolic constituents. Many studies have been carried out in coffee, such as the optimum condition and extraction methods have been well explored, to attain maximum bioactive constituents with antioxidant potency from their raw materials (Boeira et al., 2018; Santos et al., 2014).

### Total Polyphenol Content and Total Flavonoid Content in Coffee Peel

**FIGURE 1:**
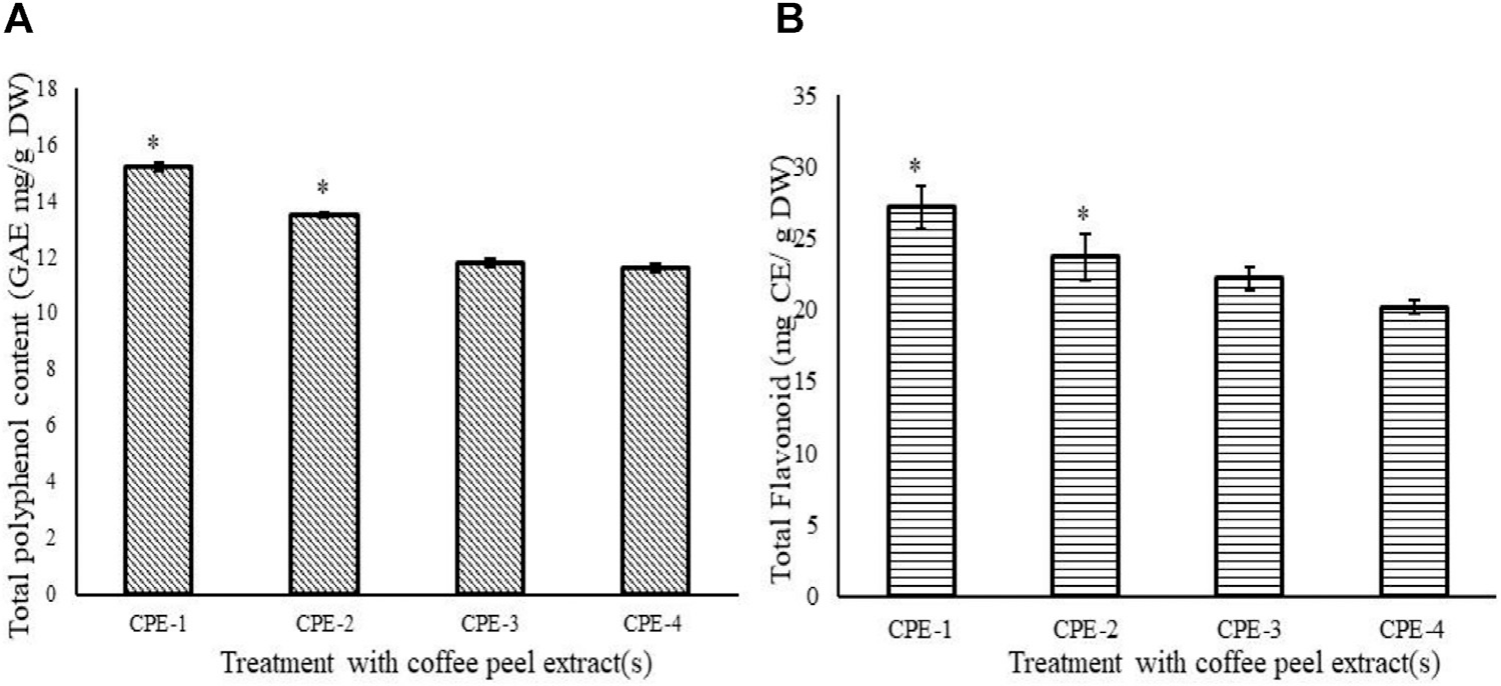
TPC (GAE mg/g DW) **(A)** and flavonoid (mg CE/g DW) **(B)** in CPEs (CPE-1 = 0.5 g, CPE-2 = 0.75 g, CPE-3 = 1 g, and CPE-4 = 1.25 g). All the values are expressed as mean ± SD and are significantly different at *p ≤ 0.05.

The TPC of the CPE has been shown in Figure 1A. A sample of CPE-1 (0.5 g CP) exhibited significantly (*p* ≤ 0.05) the highest amount of polyphenol. An inverse relationship has been observed between the TPC and the concentration of CP. That is, the sample containing the least concentration of CP exhibited the highest TPC (CPE-1 > CPE-2 > CPE-3 > CPE-4). The TFC of the CPE has been shown in Figure 1B. A similar pattern of TPC has been observed in TFC also. Likewise, an inverse relationship has been observed between TFC and the concentration of CP. That is, the sample containing the least concentration of CP showed the highest TFC (CPE-1 > CPE-2 > CPE-3 > CPE-4). A significant (*p* ≤ 0.05) difference in TFC has been observed between the samples except between CPE-3 and CPE-4. In this study, the TPC values ranged from 11.65 to 15.23 GAE mg/g CP and the TFC values ranged from 20.24 to 27.22 mg catechin equivalent. Our results are in line with those of Alkaltham et al. (2020). They reported the comparison of the TPC and TFC of coffee fruit beans, pulp, and parchment; the TPC was 0.392, 0.183, and 0.110 mg GAE/g DW, respectively, and the TFC was 30.30, 8.02, and 0.638 mg CE/g DW, respectively. In this context, Silva et al. (2021) have reported that comparison of the coffee husk extracted with different organic solvents and techniques with the conventional extraction method (water bath) exhibited the TPC and TFC that ranged from 31.35 to 97.89 mg CAE/g and 0.63–9.93 mg CE/g, respectively, and ultrasound-assisted extraction method exhibited TPC and TFC ranging from 16.54 to 90.95 and 0.21–15.69, respectively. Silva et al. (2021) confirmed that water: ethanol mixture was the extracting solution with a higher potential, and dehydration was a very significant factor to concentrate and provided higher levels of phenolic compounds. Andrade et al. (2012) reported that TPC ranged from 16.1 mg CAE/g to 423 mg CAE/g for coffee husk. The difference in results might be due to the differences in the variety and the extraction methods and extricating solvents used.

### Quantification of Phenolic Compound in Coffee Peel Extracts Using HPLC

Individual polyphenols from coffee extracts (CPE-1, CPE-2, CPE-3, and CPE-4) were separated by HPLC and determined quantitatively. The major phenolic compounds which were found in CPE are presented in Table 1, and the results are expressed in mg/g.

**TABLE 1:** Results of the phenolic compounds of CPEs (mg/100 g).

Sample	Chlorogenic acid (mg/100 g)	Vanillin (mg/100 g)	Salicylic acid (mg/100 g)
CPE-1	741.2	365.6	68.0
CPE-2	692.3	322.7	61.3
CPE-3	871.0	409.6	79.6
CPE-4	589.9	301.8	49.8

CPE-1, 0.5 g; CPE-2, 0.75 g; CPE-3, 1 g; and CPE-4, 1.25 g.

**FIGURE 2:**
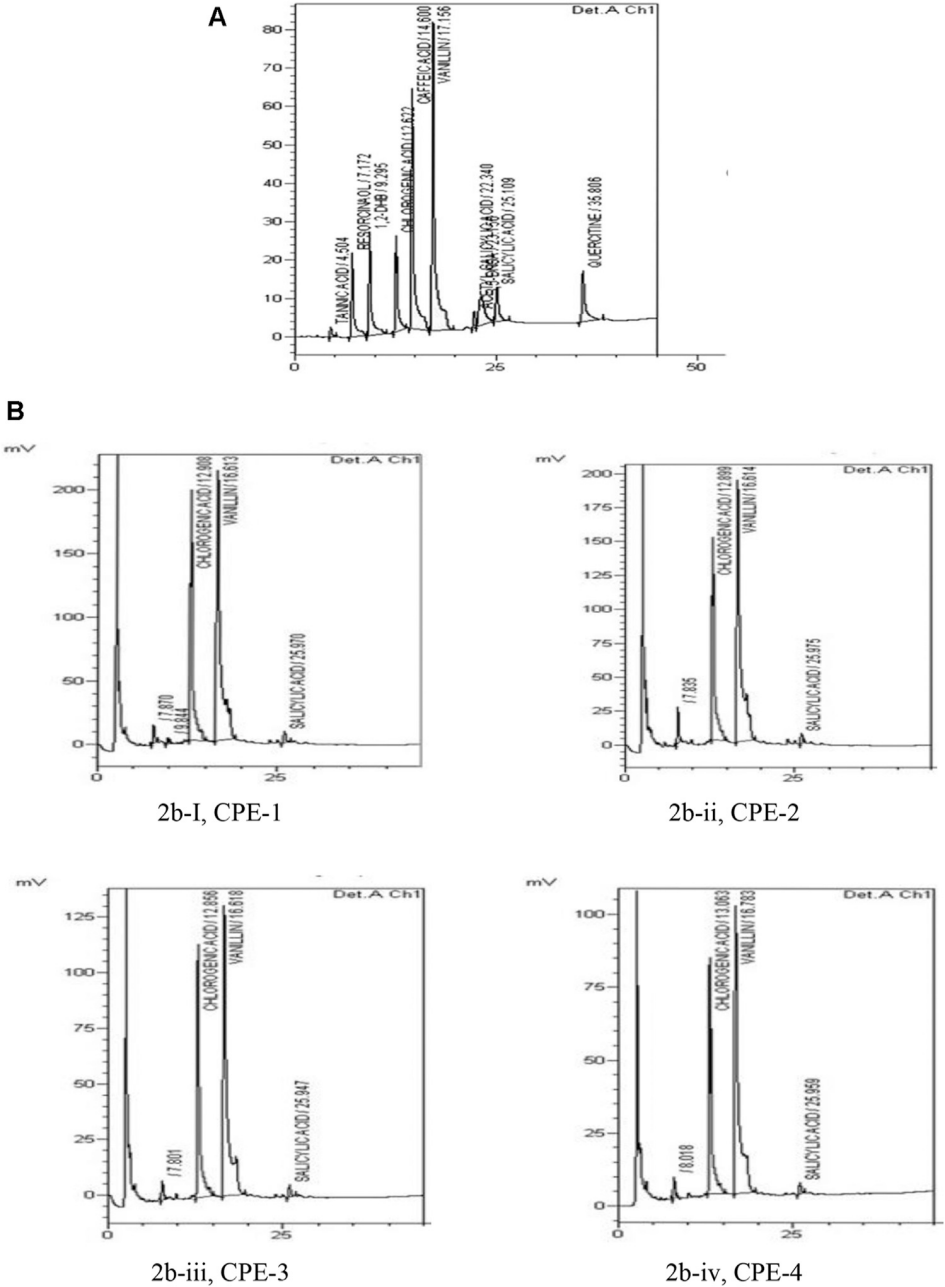
Chromatogram of standard phenolic compounds **(A)** and chromatogram of samples **(B)**, such as CPE-1 = 0.5 g **(Bi)**, CPE-2 = 0.75 g **(Bii)**, CPE-3 = 1 g **(Biii)**, and CPE-4 = 1.25 g **(Biv)**.

Chromatograms of the standard phenolic compounds and samples have been presented in Figures 2A,B, respectively. It has been found that the amount of CGA present in 100 g of CPE ranges from 589.9 to 871.0 mg. The highest amounts of CGA present in CP are as follows: CPE-3 > CPE-1 > CPE-2 > CPE-4 (Table 1). The observed results confirmed that there was no significant correlation between the CGA quantity and the increasing amount of CP [CPE-1 (0.5 g), CPE-2 (0.75 g), CPE-3 (1 g), and CPE-4 (1.25 g)] taken for extraction. CPE-3 (1 g of CP) possessed a higher amount of phenolic compounds, such as CGA (871.0 mg/100 g), vanillin (409.6 mg/100 g), and salicylic acid (79.6 mg/100 g). As mentioned in Table 1, the average quantitative data showed that the CGA which is acknowledged for its antioxidant capacities ranged from 589.9 to 871.2 mg/100 g phenolic compound. The amount of vanillin ranged from 301.8 to 409.6 mg/100 g phenolic compound, and salicylic acid was the lowest (79.6–49.8 mg/100 g phenolic compound) among all. In this context, Jaiswal et al. (2012) have reported that the different roasting methods of coffee seeds in the laboratory consist of 40–209 mg/100 g in *Coffea arabica* and 40–509 mg/100 g in *Coffea canephora* seeds. In general, the level of CGA in coffee brews varies largely from 26 mg/100 ml to the extreme of 1141 mg/100 ml in different countries (Farah and de Paula, 2019). The amount of CGA differs due to various factors such as the species, the degree of maturation, the altitude, and the presence or absence of shade, as well as resistance to some diseases (Aerts and Baumann, 1994). According to our observation, the quantity of the CGA range in CP was found to be 589.9–871.0 mg/100 g in comparison with that in coffee seeds which varied from 200 to 301 mg/100 g. CGA is the main phenolic compound present in coffee beans that possesses antioxidant properties (Andrade et al., 2012; Clifford et al., 2017; Alkaltham et al., 2020). However, the CP contains a rich amount of polyphenols CGA along with total flavonoid, vanillin, and salicylic acid which favors effective inhibition of oxidative stress–associated cellular senescence and DNA damage (Xu et al., 2012; Cinkilic et al., 2013; Ameca et al., 2018).

### Carbohydrate Hydrolysis Enzymatic α-Amylase Assay

Starch is one of the main sources of dietary energy which is mainly digested in the gastrointestinal tract by pancreatic α-amylase. Rate of starch digestion and absorption may help to control postprandial hyperglycemia, and so in diabetics, decelerating the digestion of starch may have a favorable effect on the glycemic index (Notkins, 2002). Hyperglycemia has been found to be linked with the threat of various diseases, such as obesity and cardiovascular- and kidney-related issues, which in turn increases the necessity for strict glycemic control (Blaak et al., 2012). In the intraluminal phase, α-A is the main enzyme accountable for starch digestion. So, an α-A inhibitor will slow down the carbohydrate digestion (Golay et al., 1991).

**FIGURE 3:**
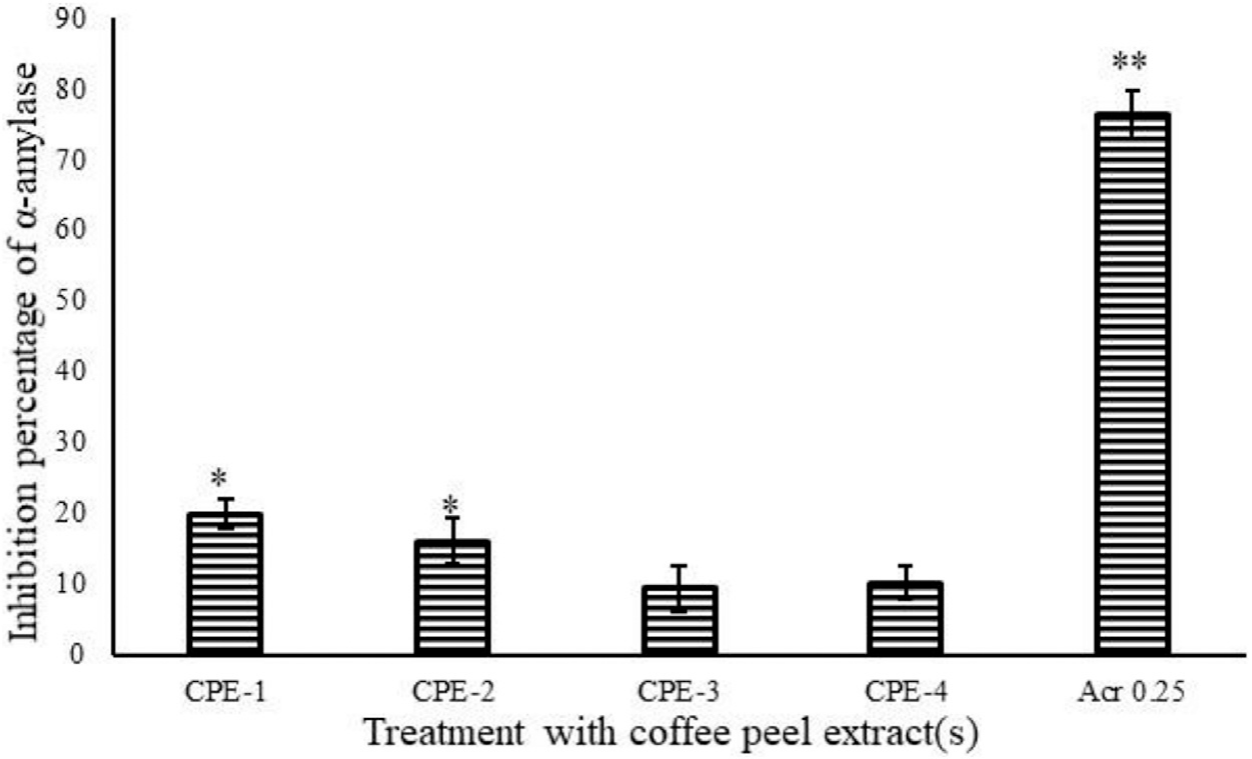
Inhibition percentage of α-A by acarbose and CPEs (CPE-1 = 0.5 g, CPE-2 = 0.75 g, CPE-3 = 1 g, and CPE-4 = 1.25 g). All the values are expressed as mean ± SD and are significantly different at *p ≤ 0.05 and **p ≤ 0.001.

Four extracts from the CP (CPE-1, CPE-2, CPE-3, and CPE-4) were tested for porcine pancreatic α-A enzyme inhibition. All extracts weakly inhibited porcine pancreatic α-A. The extract CPE-1 (0.5 g) provided the highest inhibitory effect on α-A (19.88%/mg extract). It was able to inhibit porcine pancreatic α-A by 20%. We observed in CPE-2, CPE-3, and CPE-4 a lesser than 20% inhibition of porcine pancreatic α-A with an insignificant (*p* ≥ 0.05) difference. IC_50_ can be defined as the lowermost concentration of a compound that is required to inhibit 50% of the enzyme activity (Oboh et al., 2015). Acarbose was able to inhibit the enzyme α-A at 70% as shown in Figure 3. For acarbose, an IC_50_ of 0.25 g/ml was determined (Figure 3). The inhibition by acarbose was higher when compared to CPE. α-A is one of the most significant digestive enzymes in humans, and it works as a catalyst in the reaction which implicates the hydrolysis of alpha-1,4 glycosidic linkages of large molecules like starch into smaller fragments of sugars, i.e., monosaccharides. The sugar level in the blood rises due to excess conversion of starch to sugar in response to which the insulin instructs cells to metabolize the surplus sugar and store them in the form of sugar. This cycle keeps on going interminably until in certain conditions where the activity of the amylase enzyme increases or a deficiency of insulin or insulin resistance occurs which in turn leads to an increase in blood sugar (Agarwal and Gupta, 2016). There are two main biochemical mechanisms related to raised blood glucose levels, and they are enzymatic (the polyol pathway) and nonenzymatic glycosylation (Creutzfeldt, 1999). Acarbose is used for treatment of diabetes, and it inhibits the activities of α-A (Jyothi et al., 2014). In this study, the observed inhibition of α-A even though it was weak might be due to the presence of CGA, which is inconsistent with earlier studies (Ranilla et al., 2010; Narita and Inouye, 2011). The inhibition of α-A by flavonoids is due to the formation of hydrogen bonds by hydroxyl groups with specific amino acids at the enzymes' active sites (De Sales et al., 2012). The extract CPE-1 (0.5 g) and CPE-2 of CP provided the highest inhibitory effect on α-A (19.88 and 16.07%/mg extract), which has been identified with the highest TPC and TF content compared to other extracts. Moreover, lower levels of TPC and TFC (µg/mg of extract) show lowest inhibition (statistically insignificant differences were observed between CP3 and CP4).

### In Vitro Cell Culture Study Using hMSCs

Cells can normally protect themselves from ROS damage through their self-defense antioxidative mechanism. But, hMSCs have less antioxidant capacity and are more sensitive to oxidative stress, when compared to differentiated lineages such as adipocytes, chondrocytes, and osteoblasts (Yagi et al., 2013). In regenerative medicine, cellular stress produces excessive ROS or exogenous addition of H_2_O_2_ might impair the capacity of differentiation to multiline ages or self-renewal and proliferation were impaired (Zou et al., 2004). Excessive free radical damage to hMSCs might end with cell senescence and arrested cell divisions (Bajek et al., 2012). Orciani et al. (2010) reported that ROS inhibits hMSCs to osteogenesis differentiation and undifferentiated hMSCs, having a higher lactate production rate and glycolytic enzyme levels. Lesser osteogenic differentiation results in bone weakness and arthritis. In contrast, ROS increases adipocyte differentiation with upregulated antioxidant gene expressions, similar to osteogenic differentiation mitochondrial biogenesis of glycolysis, and the lactate level increased in adipogenesis (Denu and Hematti, 2016).

**FIGURE 4:**
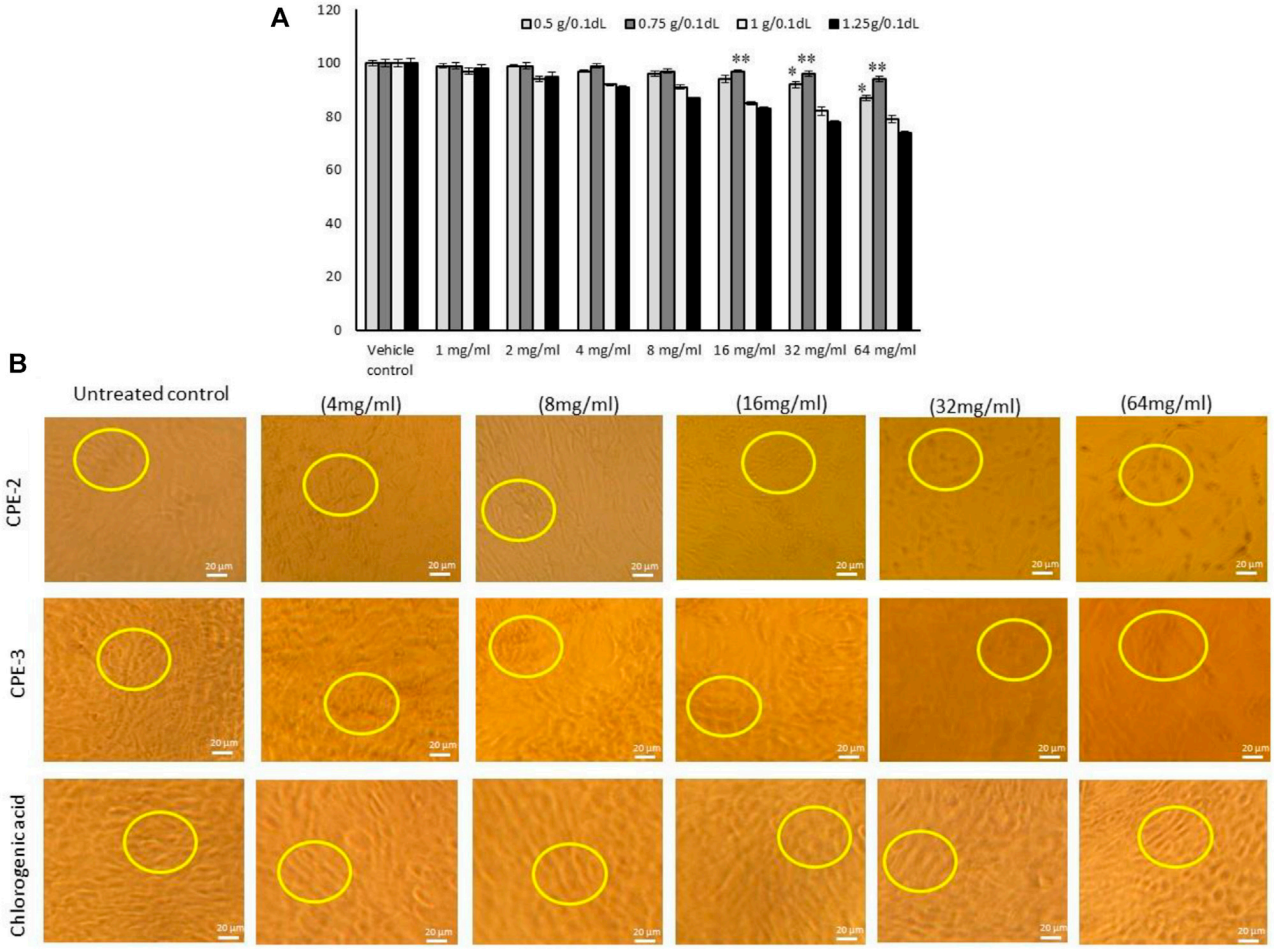
In vitro cytotoxic effect **(A)** and light microscopy images for cell morphology **(B)** in hMSCs treated with increasing concentration of CPEs. All the images were captured with ×10 magnification. CPE-1 = 0.5 g, CPE-2 = 0.75 g, CPE-3 = 1 g, and CPE-4 = 1.25 g for 48 h. Each of the values are mean ± SD (*n* = 6). *p ≤ 0.05 and **p ≤ 0.001 by comparison with vehicle control.

The quantified rich TPC and TFC of CPE favors the antioxidant and oxidative stress quenching capacity. It was confirmed by the present study that increasing the concentration of CPE treated to hMSCs resulted in increased cell proliferation and viability in CPE-2 (94%) when compared to the other extracts [CPE-3 (79%) or CPE-4 (74%)] (Figure 4). In addition, CPE-2 showed significantly (*p* ≤ 0.05) increased cell viability (85%) against the increasing concentration of extract treatment. It may be due to the availability of rich amounts of phenolic and flavonoid components in CPE-2. The observed results were compared with the reference drug, CGA. In this context, Chen et al. (2008) have reported that the presence of antioxidant polyphenols, such as epigallocatechin and tocopherol, protects the hMSCs from oxidative stress and increased proliferation capacity. Phenolic compounds and phenols are widely distributed from natural agents, so they have been considered as powerful agents for their antioxidative activities in hMSCs.

**FIGURE 5:**
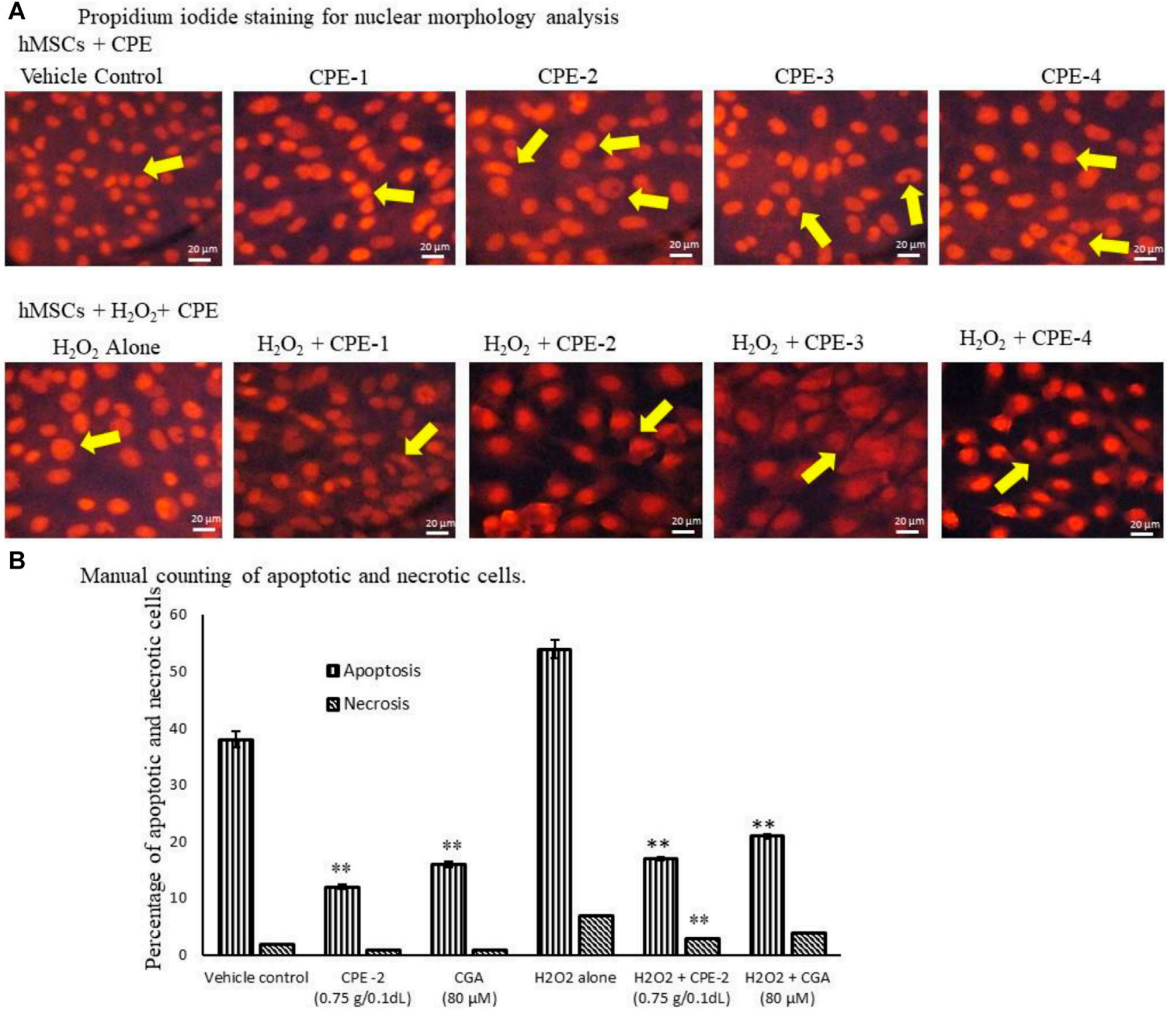
Analysis of vehicle control and CPEs–treated hMSCs nuclear morphology by PI Staining for nuclear damage **(A)** and manual counting for apoptotic or necrosis cells **(B)** after 48 h. All the images were captured with ×20 magnification. CPE-1 = 0.5 g, CPE-2 = 0.75 g, CPE-3 = 1 g, and CPE-4 = 1.25 g. Each of the values are mean ± SD (*n* = 6). *p ≤ 0.05 and **p ≤ 0.001 were compared with vehicle control or H_2_O_2_ alone treated hMSCs.

In propidium iodide (PI) staining analysis, CPE-2–treated cells were compared with CGA-treated cells; only 3% of the cells showed a nuclear morphology change in CPE-2 treatment, but CGA (80 μM) treatment showed 11% of different nuclear morphology (Figure 5A). H_2_O_2_-induced oxidative stressed hMSCs were treated with TPC- and TFC-rich CPE-2, with very less number (7%) of nuclear damaged cells, and H_2_O_2_ + CGA–treated cells showed 16% of morphologically different cells that were observed in PI nuclear staining. But, H_2_O_2_ alone treated cells showed 27% of pyknosis, and chromatin condensation was observed under an inverted microscope manually (Figure 5B). The observed results are in line with the previous reports that higher ROS levels cause cellular damage and dysfunction. The accumulation of ROS can damage cellular DNA, glycoproteins, and glycolipids. Human patients with atherosclerosis and diabetes have been identified with elevated oxidative stress and reduced capacity to inhibit T-cell proliferation (Mancini et al., 2015). Previously, Cha et al. (2014) have reported that CGA effectively protects oxidative stress induced by DNA damage in human keratinocytes.

**FIGURE 6:**
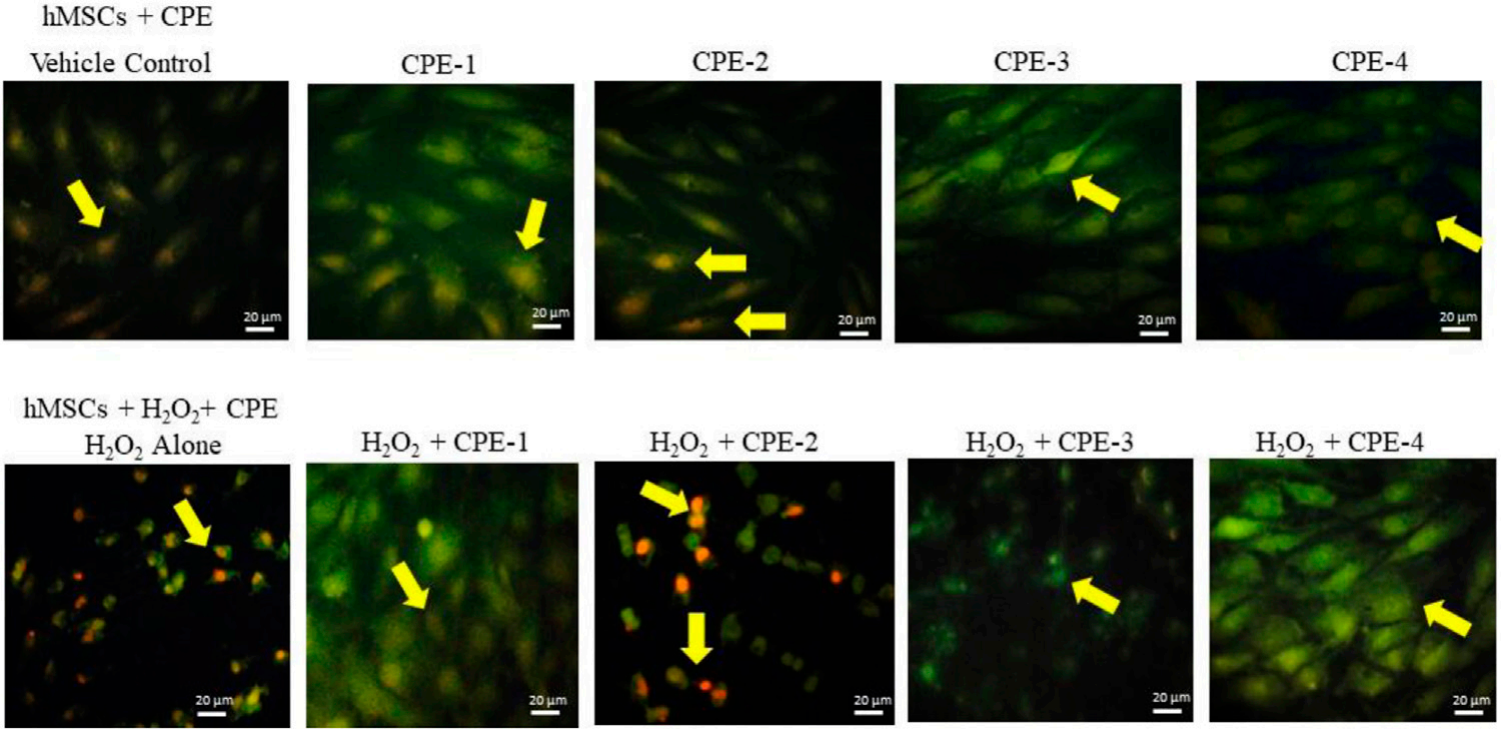
Analysis of mitochondrial membrane potential using JC-1 staining for vehicle control and CPEs–treated hMSCs after 48 h. All the images were captured with ×20 magnification (CPE-1 = 0.5 g, CPE-2 = 0.75 g, CPE-3 = 1 g, and CPE-4 = 1.25 g).

Alterations in mitochondrial electrochemical gradient and transmembrane potential majorly affect the cellular ATP production progress. The mitochondria use oxidizable substrates majorly to produce an electrochemical proton gradient across the mitochondrial membrane (De Mello et al., 2018). Mitochondrial oxidative stress alters the inner membrane polarity and leads to a loss of mitochondrial transmembrane potential (Δψ_m_) further deregulating mitochondrial electronegative and transport capacity (Zorova et al., 2018). The internal electronegative organelle of the mitochondria promotes internal uptake of cations and outward transport of anions (ATP). During excessive ROS or free radicals, the mitochondrial permeability process decreases and a loss of the electrochemical gradient which is the major indicator for mitochondrial function and cell health occurs (Sivandzade et al., 2019). Regulation of energy homeostasis has been majorly controlled by the mitochondria, and mitochondrial imbalance has been associated with the development of obesity (Picard et al., 2011). Polyphenols from plant sources have been described as antioxidants; they have the potential to eliminate ROS and free radicals, which normalize the mitochondrial membrane potential (Robb et al., 2017). In the present study, a rich amount of polyphenols and flavonoids in CPE-2 significantly enhanced the mitochondrial health when compared to the other extracts (Figure 6). It has been confirmed by the healthy electronegative mitochondria uptake of the cationic natural green florescent JC-1 dye, which was further converted internally into an irreversible red florescent J-aggregate. CPE-1 also showed moderate J-aggregate accumulation in oxidative stressed hMSCs; this effect was not observed in normal hMSCs. But, in CPE-3 and CPE-4, the conversion of J-aggregates from JC-1 was not observed both in normal as well as in oxidative stressed hMSCs, confirming the unhealthy mitochondria with a decreased mitochondrial membrane potential and membrane leakage.

**FIGURE 7:**
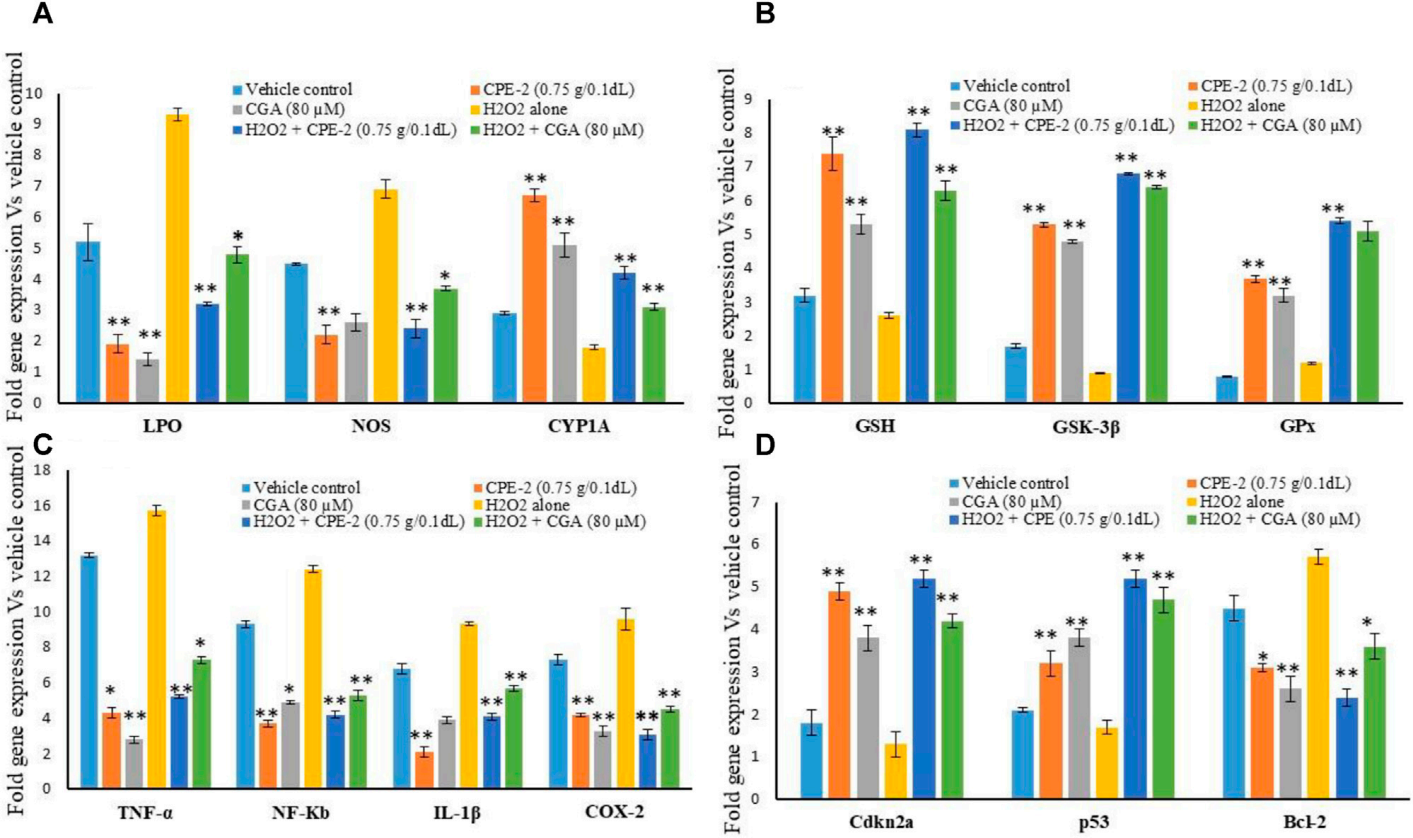
Effect of vehicle control and CPEs (CPE-2; 0.75 g/0.1 dl) on oxidative stress **(A)**, antioxidant **(B)**, pro-inflammatory **(C),** and tumor suppression **(D)** related gene expression levels after 48 h. Each of the values are mean ± SD (*n* = 6). *p ≤ 0.05 and **p ≤ 0.001 were compared with vehicle control or H_2_O_2_ alone treated hMSCs.

In addition, changes in mRNA expression levels in oxidative stress, and immunomodulatory and tumor suppressive-related genes in normal and H_2_O_2_-induced oxidative stressed hMSCs after polyphenol rich CP treatment were observed. The results confirmed that the increased antioxidant and mitochondrial membrane potential capacity of CPE-2 significantly (*p* ≤ 0.001) decreased the oxidative stress markers of LPS and NOX-2 when compared to the untreated or H_2_O_2_ alone treated hMSCs (Figure 7A). The effect was observed in CGA-treated hMSCs with a significant level of *p* ≤ 0.05. The expression level of CYP1A was significantly (*p* ≤ 0.001) increased in CPE-2 treatment, and it was supported by the increased mitochondrial health (in JC-1 assay) after CPE treatment. The antioxidant potential of CPE-2 treatment was confirmed by the increased expression levels of GSH, GSK-3β, and GPx when compared to those of vehicle control or oxidative stress–induced hMSCs (Figure 7B). In regenerative medicine, due to the multipotent differentiation potential of the hMSCs, these are implanted for tissue loss and contribute to tissue repair with suppressing inflammatory rejection (Di Nicola et al., 2002). After the generation of excessive ROS or exogenous H_2_O_2_ at the ischemic site, the transplanted MSCs might impair self-proliferation and multilineage capacity (Choo et al., 2014). The present finding confirmed the increased expression of antioxidant genes that was associated with quenching of oxidative stress aid to overcome the impairment of multiline age capacity of hMSCs at the graft site.

Pro-inflammatory cytokines and cellular metabolic inflammation–related gene expression levels also significantly (*p* ≤ 0.001) decreased in CPE-2–treated cells. TNF-α and NF-κb expression levels were observed twofold higher in oxidative stressed hMSCs. IL-1β and COX-2 expressions were significantly increased to a fold in untreated and oxidative stressed cells when compared to the CPE-2–treated cells (Figure 7C). In addition, tumor suppressor-related genes cdkn2a and p53 expressions were significantly decreased twofold when compared to oxidative stressed hMSCs. BCL-2 expression was decreased in CPE-2–treated and CGA-treated hMSCs both in normal and H_2_O_2_-induced oxidative stressed hMSCs (Figure 7D). In response to diverse stresses such as DNA damage or hypoxia, the tumor suppressor p53 is accumulated and cellular proliferation arrested (Subash-Babu et al., 2017). Upon oxidative stress or hyperproliferation signaling conditions, mdm2 negatively regulates p53 which leads to transition from the resting phase (G1) to DNA synthesis phase (S), and subsequently, cancer cell progression continues. In unstressed cells, p53 is tightly regulated by murine double minute 2 (MDM2) by maintaining p53 at low levels. The cell-cycle gatekeeper gene p^14ARF^ neutralizing mdm2 function *via* cyclin-dependent protein kinase (Cdkn2A) leads to increased levels of active p53 (Chin et al., 1998).

External stimulus of oxidative stress or cellular stress causes DNA damage or cellular senescence. CP has the potential to overcome hypoxia and oxidative stress, therefore, it retains bioactive, secondary metabolites such as phenolic compounds, alkaloids, and flavonoid compounds. We identified CPE having a rich amount of polyphenol-CGA, vanillin, and salicylic acid. External stimulus of oxidative stress to hMSCs regulates stress-induced DNA damage, cell senescence, and impaired multilineage. But, CPE treatment to stressed hMSCs effectively downregulated the pro-oxidant LPO and NOX2 and enhanced the antioxidant mRNA. In addition, CP treated to stressed hMSCs potentially downregulated pro-inflammatory cytokines and enhanced tumor suppressor expressions, which is more beneficial for the cellular multilineage, such as osteogenic differentiation and cellular regeneration progress. In this context, several plant-derived compounds were found to fight against a wide range of cancer models, such as the colon, breast, liver, and prostate cancer suppression models (Wang, et al., 2012).

## Conclusion

The present findings confirmed that the presence of total polyphenol-CGA, vanillin, and salicylic acid in CPE-2 (0.75g/10 ml) effectively inhibits pancreatic α-A inhibition and arrests H_2_O_2_-induced oxidative stress that may be due to the antioxidative nature of polyphenol and flavonoids. The antioxidant capacity of CP quenches the oxidative stress in hMSCs which decreases mitochondrial oxidative stress. Overall, the oxidative stress, and pro-inflammatory and tumor suppressor gene expression levels were normalized in CPE-treated oxidative stressed hMSCs. The present study confirmed that CP-derived polyphenol and flavonoid effectively quench the oxidative stress in hMSCs, which support to protect themselves from free radical or ROS damage through its self-defensive antioxidative mechanism during cellular multilineage and regenerative therapy.

## Data Availability

The raw data supporting the conclusions of this article will be made available by the authors, without undue reservation.

## References

[B1] Aerts R. J. Baumann T. W. (1994). Distribution and Utilization of Chlorogenic Acid inCoffeaseedlings, J. Exp. Bot. 45, 497–503. 10.1093/jxb/45.4.497

[B2] Agarwal P. Gupta R. (2016). Alpha-amylase Inhibition Can Treat Diabetes Mellitus, Res. Rev. J. Med. Health Sci. 5, 1–9.

[B3] Alkaltham M. S. Salamatullah A. Hayat K. (2020), Determination of Coffee Fruit Antioxidants Cultivated in Saudi Arabia under Different Drying Conditions, Food Measure 14, 1306–1313. 10.1007/s11694-020-00378-4

[B4] Ameca G. M. Cerrilla M. E. O. Córdoba P. Z. Cruz A. D. Hernández M. S. Haro J. H. (2018). Chemical Composition and Antioxidant Capacity of Coffee Pulp, Ciênc. Agrotec. 42, 307–313. 10.1590/1413-70542018423000818

[B5] Andrade K. S. Gonçalvez R. T. Maraschin M. Ribeiro-Do-Valle R. M. Martínez J. Ferreira S. R. (2012). Supercritical Fluid Extraction from Spent Coffee Grounds and Coffee Husks: Antioxidant Activity and Effect of Operational Variables on Extract Composition, Talanta 88, 544–552. 10.1016/j.talanta.2011.11.031 22265539

[B6] Bajek A. Czerwinski M. Olkowska J. Gurtowska N. Kloskowski T. Drewa T. (2012). Does Aging of Mesenchymal Stem Cells Limit Their Potential Application in Clinical Practice?, Aging Clin. Exp. Res. 24, 404–411. 10.3275/8424 22595834

[B7] Bhupathiraju S. N. Pan A. Malik V. S. Manson J. E. Willett W. C. van Dam R. M. (2013). Caffeinated and Caffeine-free Beverages and Risk of Type 2 Diabetes, Am. J. Clin. Nutr. 97, 155–166. 10.3945/ajcn.112.048603 23151535 PMC3522135

[B8] Blaak E. E. Antoine J. M. Benton D. Björck I. Bozzetto L. Brouns F. (2012). Impact of Postprandial Glycaemia on Health and Prevention of Disease, Obes. Rev. 13, 923–984. 10.1111/j.1467-789X.2012.01011.x 22780564 PMC3494382

[B9] Boeira C. P. Piovesan N. Soquetta M. B. Flores D. C. B. Lucas B. N. Barin J. S. (2018). Ultrasonic Assisted Extraction to Obtain Bioactive, Antioxidant and Antimicrobial Compounds from Marcela, Cienc. Rural 48, 1–6. 10.1590/0103-8478cr20170772

[B10] Cano-Marquina A. Tarín J. J. Cano A. (2013). The Impact of Coffee on Health, Maturitas 75, 7–21. 10.1016/j.maturitas.2013.02.002 23465359

[B11] Cha J. W. Piao M. J. Kim K. C. Yao C. W. Zheng J. Kim S. M. (2014). The Polyphenol Chlorogenic Acid Attenuates UVB-Mediated Oxidative Stress in Human HaCaT Keratinocytes, Biomol. Ther. (Seoul) 22, 136–142. 10.4062/biomolther.2014.006 24753819 PMC3975475

[B12] Chen C. T. Shih Y. R. Kuo T. K. Lee O. K. Wei Y. H. (2008). Coordinated Changes of Mitochondrial Biogenesis and Antioxidant Enzymes during Osteogenic Differentiation of Human Mesenchymal Stem Cells, Stem Cells 26, 960–968. 10.1634/stemcells.2007-0509 18218821

[B13] Chin L. Pomerantz J. DePinho R. A. (1998). The INK4a/ARF Tumor Suppressor: One Gene-Ttwo Products-Ttwo Pathways, Trends Biochem. Sci. 23, 291–296. 10.1016/s0968-0004(98)01236-5 9757829

[B14] Choo K. B. Tai L. Hymavathee K. S. Wong C. Y. Nguyen P. N. Huang C. J. (2014). Oxidative Stress-Induced Premature Senescence in Wharton's Jelly-Derived Mesenchymal Stem Cells, Int. J. Med. Sci. 11, 1201–1207. 10.7150/ijms.8356 25249788 PMC4166865

[B15] Cinkilic N. Cetintas S. K. Zorlu T. Vatan O. Yilmaz D. Cavas T. (2013). Radioprotection by Two Phenolic Compounds: Chlorogenic and Quinic Acid, on X-ray Induced DNA Damage in Human Blood Lymphocytes *In Vitro* , Food Chem. Toxicol. 53, 359–363. 10.1016/j.fct.2012.12.008 23266271

[B16] Clifford M. N. Jaganath I. B. Ludwig I. A. Crozier A. (2017). Chlorogenic Acids and the Acyl-Quinic Acids: Discovery, Biosynthesis, Bioavailability and Bioactivity, Nat. Prod. Rep. 34, 1391–1421. 10.1039/C7NP00030H 29160894

[B17] Creutzfeldt W. (1999). Effects of the Alpha-Glucosidase Inhibitor Acarbose on the Development of Long-Term Complications in Diabetic Animals: Pathophysiological and Therapeutic Implications, Diabetes Metab. Res. Rev. 15, 289–296. 10.1002/(sici)1520-7560(199907/08)15:4<289:aid-dmrr48>3.0.co;2-v 10495478

[B18] De Mello A. H. Costa A. B. Engel J. D. G. Rezin G. T. (2018). Mitochondrial Dysfunction in Obesity, Life Sci. 192, 26–32. 10.1016/j.lfs.2017.11.019 29155300

[B19] Denu R. A. Hematti P. (2016). Effects of Oxidative Stress on Mesenchymal Stem Cell Biology, Oxid Med. Cel Longev 2016, 1-9. 10.1155/2016/2989076 PMC4928004 27413419

[B20] Devine S. M. Bartholomew A. M. Mahmud N. Nelson M. Patil S. Hardy W. (2001). Mesenchymal Stem Cells Are Capable of Homing to the Bone Marrow of Non-human Primates Following Systemic Infusion, Exp. Hematol. 29, 244–255. 10.1016/S0301-472X(00)00635-4 11166464

[B21] Di Nicola M. Carlo-Stella C. Magni M. Milanesi M. Longoni P. D. Matteucci P. (2002). Human Bone Marrow Stromal Cells Suppress T-Lymphocyte Proliferation Induced by Cellular or Nonspecific Mitogenic Stimuli, Blood 99, 3838–3843. 10.1182/blood.v99.10.3838 11986244

[B22] Durak A. Gawlik-Dziki U. Pecio L. (2014). Coffee with Cinnamon - Impact of Phytochemicals Interactions on Antioxidant and Anti-inflammatory *In Vitro* Activity, Food Chem. 162, 81–88. 10.1016/j.foodchem.2014.03.132 24874360

[B23] Faostat Food and Agriculture Organization of the United Nations, Available online: http://www.fao.org/faostat/en/#data/QC (Accessed April 30, 2021).

[B24] Farah A. dePaula Lima J. (2019). Consumption of Chlorogenic Acids through Coffee and Health Implications, Beverages 5, 11. 10.3390/beverages5010011

[B25] Farah A. Donangelo C. M. (2006). Phenolic Compounds in Coffee, Braz. J. Plant Physiol. 18, 23–36. 10.1590/s1677-04202006000100003

[B26] Fujioka K. Shibamoto T. (2006). Quantitation of Volatiles and Nonvolatile Acids in an Extract from Coffee Beverages: Correlation with Antioxidant Activity, J. Agric. Food Chem. 54, 6054–6058. 10.1021/jf060460x 16881716

[B27] Golay A. Schneider H. Temler E. Felber J. P. (1991). Effect of Trestatin, an Amylase Inhibitor, Incorporated into Bread, on Glycemic Responses in normal and Diabetic Patients, Am. J. Clin. Nutr. 53, 61–65. 10.1093/ajcn/53.1.61 1701612

[B28] Hasenah A. Houghton P. J. Soumyanath A. (2006). α -Amylase Inhibitory Activity of Some Malaysian Plants Used to Treatdiabetes; with Particular Reference to Phyllanthus Amarus, J. Ethno Pharma 107, 449–455. 10.1016/j.jep.2006.04.004 16678367

[B29] Hayat K. Abbas S. Jia C. Xia S. Zhang X. (2011). Comparative Study on Phenolic Compounds and Antioxidant Activity of Feutrell's Early and Kinnow Peel Extracts. J. Food Biochem. 35, 454–471. 10.1111/j.1745-4514.2010.00395.x

[B30] Hayat K. (2020). Impact of Drying Methods on the Functional Properties of Peppermint (Mentha Piperita L.) Leaves. Sci. Lett. J. 8, 36–42. 10.1111/j.1745-4514.2010.00395.x

[B31] Hečimović I. Belščak-Cvitanović A. Horžić D. Komes D. (2011). Comparative Study of Polyphenols and Caffeine in Different Coffee Varieties Affected by the Degree of Roasting, Food Chem. 129, 991–1000. 10.1016/j.foodchem.2011.05.059 25212328

[B32] Ho C. H. L. Cacace J. E. Mazza G. (2008). Mass Transfer during Pressurized Low Polarity Water Extraction of Lignans from Flaxseed Meal, J. Food Eng. 89, 64–71. 10.1016/j.jfoodeng.2008.04.003

[B33] Honczarenko M. Le Y. Swierkowski M. Ghiran I. Glodek A. M. Silberstein L. E. (2006). Human Bone Marrow Stromal Cells Express a Distinct Set of Biologically Functional Chemokine Receptors, Stem cells 24, 1030–1041. 10.1634/stemcells.2005-0319 16253981

[B34] Jaiswal R. Matei M. F. Golon A. Witt M. Kuhnert N. (2012). Understanding the Fate of Chlorogenic Acids in Coffee Roasting Using Mass Spectrometry Based Targeted and Non-targeted Analytical Strategies, Food Funct. 3, 976–984. 10.1039/C2FO10260A 22833076

[B35] Janissen B. Huynh T. (2018). Chemical Composition and Value-Adding Applications of Coffee Industry By-Products: A Review, Resour. Conservation Recycling 128, 110–117. 10.1016/j.resconrec.2017.10.001

[B36] Jyothi K. S. N. Shailaja M. Viveni J. Suresh C. (2014). Identification of a Proteinaceous Alpha Amylase Inhibitor from a Medicinal Herb Oxalis Corniculata L. (Oxalidaceae), J. Homeop Ayurv Med. 3, 165.

[B37] Kähkönen M. P. Hopia A. I. Vuorela H. J. Rauha J. P. Pihlaja K. Kujala T. S. (1999). Antioxidant Activity of Plant Extracts Containing Phenolic Compounds, J. Agric. Food Chem. 47, 3954–3962. 10.1021/jf990146l 10552749

[B38] Kizilay Mancini O. Shum-Tim D. Stochaj U. Correa J. A. Colmegna I. (2015). Age, Atherosclerosis and Type 2 Diabetes Reduce Human Mesenchymal Stromal Cell-Mediated T-Cell Suppression, Stem Cel Res Ther 6, 140. 10.1186/s13287-015-0127-9 PMC4529693 26253429

[B39] Kobayashi C. I. Suda T. (2012). Regulation of Reactive Oxygen Species in Stem Cells and Cancer Stem Cells, J. Cel Physiol 227, 421–430. 10.1002/jcp.22764 21448925

[B40] Leite M. Quinta-Costa M. Leite P. S. Guimarães J. E. (1999). Critical Evaluation of Techniques to Detect and Measure Cell Death-Sstudy in a Model of UV Radiation of the Leukaemic Cell Line HL60, Anal Cel Pathol 19, 139–151. 10.1155/1999/176515 PMC4618583 10866276

[B41] Liang N. Kitts D. D. (2016). Role of Chlorogenic Acids in Controlling Oxidative and Inflammatory Stress Conditions, Nutrients 8, 16. 10.3390/nu8010016 PMC4728630 26712785

[B42] Murthy P. S. Madhava Naidu M. (2012). Sustainable Management of Coffee Industry By-Products and Value Addition-A Review, Resour. Conservation Recycling 66, 45–58. 10.1016/j.resconrec.2012.06.005

[B43] Narita Y. Inouye K. (2011). Inhibitory Effects of Chlorogenic Acids from green Coffee Beans and Cinnamate Derivatives on the Activity of Porcine Pancreas α-amylase Isozyme I, Food Chem. 127, 1532–1539. 10.1016/j.foodchem.2011.02.013

[B44] Notkins A. L. (2002). Immunologic and Genetic Factors in Type 1 Diabetes, J. Biol. Chem. 277, 43545–43548. 10.1074/jbc.R200012200 12270944

[B45] Oboh G. Agunloye O. M. Adefegha S. A. Akinyemi A. J. Ademiluyi A. O. (2015). Caffeic and Chlorogenic Acids Inhibit Key Enzymes Linked to Type 2 Diabetes (*In Vitro*): A Comparative Study, J. Basic Clin. Physiol. Pharmacol. 26, 165–170. 10.1515/jbcpp-2013-0141 24825096

[B46] Orciani M. Gorbi S. Benedetti M. Di Benedetto G. Mattioli-Belmonte M. Regoli F. (2010). Oxidative Stress Defense in Human-Skin-Derived Mesenchymal Stem Cells versus Human Keratinocytes: Different Mechanisms of protection and Cell Selection, Free Radic. Biol. Med. 49, 830–838. 10.1016/j.freeradbiomed.2010.06.007 20541604

[B47] Pari L. Karthikesan K. Menon V. P. (2010). Comparative and Combined Effect of Chlorogenic Acid and Tetrahydrocurcumin on Antioxidant Disparities in Chemical Induced Experimental Diabetes, Mol. Cel Biochem 341, 109–117. 10.1007/s11010-010-0442-5 20339905

[B48] Picard M. Taivassalo T. Gouspillou G. Hepple R. T. (2011). Mitochondria: Isolation, Structure and Function, J. Physiol. 589, 4413–4421. 10.1113/jphysiol.2011.212712 21708903 PMC3208215

[B49] Prihadi A. R. Maimulyanti A. Mellisani B. Nurhasanah N. (2020). Antioxidant Activity, Tannin Content and Dietary Fiber from Coffee Husk Extract and Potential for Nutraceutical, Rjc 12, 955–959. 10.31788/rjc.2020.1325613

[B50] Ranilla L. G. Kwon Y. I. Apostolidis E. Shetty K. (2010). Phenolic Compounds, Antioxidant Activity and *In Vitro* Inhibitory Potential against Key Enzymes Relevant for Hyperglycemia and Hypertension of Commonly Used Medicinal Plants, Herbs and Spices in Latin America, Bioresour. Technol. 101, 4676–4689. 10.1016/j.biortech.2010.01.093 20185303

[B51] Robb E. L. Moradi F. Maddalena L. A. Valente A. J. F. Fonseca J. Stuart J. A. (2017). Resveratrol Stimulates Mitochondrial Fusion by a Mechanism Requiring Mitofusin-2, Bioche Biophys. Res. Commun. 485, 249–254. 10.1016/j.bbrc.2017.02.10 28235489

[B52] Sales P. M. Souza P. M. Simeoni L. A. Silveira D. Silveira D. (2012). α-Amylase Inhibitors: a Review of Raw Material and Isolated Compounds from Plant Source, J. Pharm. Pharm. Sci. 15, 141–183. 10.18433/j35s3k 22365095

[B53] Santos J. Oliveira M. B. P. P. Ibáñez E. Herrero M. (2014). Phenolic Profile Evolution of Different Ready-To-Eat Baby-Leaf Vegetables during Storage, J. Chromatogr. A. 1327, 118–131. 10.1016/j.chroma.2013.12.085 24438834

[B54] Silva M. D. O. Honfoga J. N. B. Medeiros L. L. D. Madruga M. S. Bezerra T. K. A. (2021). Obtaining Bioactive Compounds from the Coffee Husk (Coffea Arabica L.) Using Different Extraction Methods, Molecules 26, 46. 10.3390/molecules26010046 PMC7795416 33374108

[B55] Sivandzade F. Bhalerao A. Cucullo L. (2019). Analysis of the Mitochondrial Membrane Potential Using the Cationic JC-1 Dye as a Sensitive Fluorescent Probe, Bio Protoc. 9, e3128. 10.21769/BioProtoc.3128 PMC6343665 30687773

[B56] Song H. Y. Jang H. W. Debnath T. Lee K. (2019). Analytical Method to Detect Adulteration of Ground Roasted Coffee, Int. J. Food Sci. Technol. 54, 256–262. 10.1111/ijfs.13942

[B57] Subash-Babu P. Alshammari G. M. Ignacimuthu S. Alshatwi A. (2017). Epoxy Clerodane Diterpene Inhibits MCF-7 Human Breast Cancer Cell Growth by Regulating the Expression of the Functional Apoptotic Genes Cdkn2A, Rb1, Mdm2 and P53, Biomed. Pharmacother. 87, 388–396. 10.1016/j.biopha.2016.12.091 28068628

[B58] Subramanian R. Asmawi M. Z. Sadikun A. (2008). *In Vitro* alpha-glucosidase and Alpha-Amylase Enzyme Inhibitory Effects of Andrographis Paniculata Extract and Andrographolide. Acta Biochim. Pol. 55, 391–398. 10.18388/abp.2008_3087 18511986

[B59] Tan P. W. Tan C. P. Ho C. W. (2011). Antioxidant Properties: Effect of Solid-To-Solvent Ratio on Antioxidant Compounds and Capacities of Pegaga (*Centella asiatica*), Int. Food Res. J. 18, 553–558.

[B60] Tapiero H. Tew K. D. Ba G. N. Mathe G. (2002). Polyphenols: Do They Play a Role in the Prevention of Human Pathologies? Biomed. Pharmacother. 56, 200–207. 10.1016/S0753-3322(02)00178-6 12109813

[B61] Thoo Y. Y. Ho S. K. Liang J. Y. Ho C. W. Tan C. P. (2010). Effect of Binary Solvent Extraction System, Extraction Time and Extraction Temperature on Phenolic Antioxidants and Antioxidant Capacity from Mengkudu (*Morinda citrifolia*), Food Chem. 120, 2290–2295. 10.1016/j.foodchem.2009.09.064

[B62] Wang H. Khor T. O. Shu L. Su Z. Y. Fuentes F. Lee J. H. (2012). Plants vs. Cancer: a Review on Natural Phytochemicals in Preventing and Treating Cancers and Their Drug Ability, Anti-cancer Agents Med. Chem. 12, 1281–1305. 10.2174/187152012803833026 PMC4017674 22583408

[B63] Wang T. Wu Y. Wang W. Zhang D. (2021). Association between Coffee Consumption and Functional Disability in Older US Adults, Br. J. Nutr. 125, 695–702. 10.1017/S0007114520003153 32778181

[B64] Xu J. G. Hu Q. P. Liu Y. (2012). Antioxidant and DNA-Protective Activities of Chlorogenic Acid Isomers, J. Agric. Food Chem. 60, 11625–11630. 10.1021/jf303771s 23134416

[B65] Yagi H. Tan J. Tuan R. S. (2013). Polyphenols Suppress Hydrogen Peroxide-Induced Oxidative Stress in Human Bone-Marrow Derived Mesenchymal Stem Cells, J. Cel Biochem 114, 1163–1173. 10.1002/jcb.24459 23192437

[B66] Yuan J. S. Reed A. Chen F. Stewart C. N. (2006). Statistical Analysis of Real-Time PCR Data, BMC bioinformatics 7, 1–12. 10.1186/1471-2105-7-85 16504059 PMC1395339

[B67] Zorova L. D. Popkov V. A. Plotnikov E. Y. Silachev D. N. Pevzner I. B. Jankauskas S. S. (2018). Mitochondrial Membrane Potential, Anal. Biochem. 552, 50–59. 10.1016/j.ab.2017.07.009 28711444 PMC5792320

[B68] Zou X. Li H. Chen L. Baatrup A. Bunger C. Lind M. (2004). Stimulation of Porcine Bone Marrow Stromal Cells by Hyaluronan, Dexamethasone and rhBMP-2, Biomaterials 25, 5375–5385. 10.1016/j.biomaterials.2003.12.041 15130722

